# Magnetic Study of CuFe_2_O_4_-SiO_2_ Aerogel and Xerogel Nanocomposites

**DOI:** 10.3390/nano11102680

**Published:** 2021-10-12

**Authors:** Alizé V. Gaumet, Francesco Caddeo, Danilo Loche, Anna Corrias, Maria F. Casula, Andrea Falqui, Alberto Casu

**Affiliations:** 1 School of Physical Sciences, Ingram Building, University of Kent, Canterbury CT2 7NH, UK; avg32@bath.ac.uk (A.V.G.); fcaddeo@physnet.uni-hamburg.de (F.C.); daniloche@tiscali.it (D.L.); a.corrias@kent.ac.uk (A.C.); 2 Department of Mechanical, Chemical and Materials Engineering and INSTM, University of Cagliari, Via Marengo 2, 09123 Cagliari, Italy; casulaf@unica.it; 3 Department of Physics “Aldo Pontremoli”, University of Milan, Via Celoria 16, 20133 Milan, Italy; andrea.falqui@unimi.it; 4 Biological and Environmental Sciences and Engineering (BESE) Division, King Abdullah University of Science and Technology (KAUST), Thuwal 23955-6900, Saudi Arabia

**Keywords:** copper ferrite, magnetic properties, sol-gel, aerogels, xerogels

## Abstract

CuFe_2_O_4_ is an example of ferrites whose physico-chemical properties can vary greatly at the nanoscale. Here, sol-gel techniques are used to produce CuFe_2_O_4_-SiO_2_ nanocomposites where copper ferrite nanocrystals are grown within a porous dielectric silica matrix. Nanocomposites in the form of both xerogels and aerogels with variable loadings of copper ferrite (5 wt%, 10 wt% and 15 wt%) were synthesized. Transmission electron microscopy and X-ray diffraction investigations showed the occurrence of CuFe_2_O_4_ nanoparticles with average crystal size ranging from a few nanometers up to around 9 nm, homogeneously distributed within the porous silica matrix, after thermal treatment of the samples at 900 °C. Evidence of some impurities of CuO and α-Fe_2_O_3_ was found in the aerogel samples with 10 wt% and 15 wt% loading. DC magnetometry was used to investigate the magnetic properties of these nanocomposites, as a function of the loading of copper ferrite and of the porosity characteristics. All the nanocomposites show a blocking temperature lower than RT and soft magnetic features at low temperature. The observed magnetic parameters are interpreted taking into account the occurrence of size and interaction effects in an ensemble of superparamagnetic nanoparticles distributed in a matrix. These results highlight how aerogel and xerogel matrices give rise to nanocomposites with different magnetic features and how the spatial distribution of the nanophase in the matrices modifies the final magnetic properties with respect to the case of conventional unsupported nanoparticles.

## 1. Introduction

Spinel ferrite nanoparticles with general formula MFe_2_O_4_ (where M is a bi-valent transition metal ion such as Mn^2+^, Ni^2+^, Co^2+^, Cu^2+^, Zn^2+^, etc.) have been the object of intense investigation due to their interesting optical, magnetic and catalytic properties for their potential application in storage devices [1], photo-catalysis [2], magnetic fluids [3], sensors [4] and biomedicine [5]. In particular, the introduction of copper in the general spinel ferrite crystal structure gives rise to a soft magnet, which has been deemed an interesting candidate for multiple applications in many diverse fields, ranging from sensors to catalysis [6,7,8]. This has led to a large number of studies in recent years, focusing on attaining an always increasing degree of control over the shape, size, distribution and phase of the copper ferrite nanoparticles. In fact, copper ferrite represents a special case among ferrites, since it displays two distinct crystalline phases, namely a high temperature disordered cubic *Fd-3m* phase (c-CuFe_2_O_4_) and an ordered tetragonal *I4I/amd* phase (t-CuFe_2_O_4_) that can be obtained during slow cooling in air [9]. The tetragonal phase arises from the collective Jahn–Teller distortion along one of the axes of the octahedral sites, which is typical for Cu^2+^ ions (d9) as a consequence of the removal of the e_g_ orbital degeneracy [10,11]. For this reason, many studies have been focused on synthesizing copper ferrite nanoparticles with a wide number of methods including thermal decomposition [12], hydrothermal [13], solvothermal [14], co-precipitation [15], electrospinning [16] and sol-gel [17], also taking into account that the desired crystalline phase might be obtained by selecting an appropriate thermal treatment. Moreover, in a recent report it has been shown that, independently of the final heat treatment, the cubic phase and tetragonal phase can be obtained by employing sol-gel and co-precipitation methods, respectively [18]. All the aforementioned synthetic methods yield nanoparticles with dimensions of several tens of nanometers and a poor degree of size homogeneity, mainly due to the aggregation and sintering of the nanoparticles during the thermal treatments needed to obtain the desired crystalline phase. In turn, this also affects the magnetic properties and makes it difficult to achieve a precise control over the different parameters at play in giving rise to the final magnetic behavior [19].

A fruitful strategy to produce crystalline magnetic nanoparticles with a controlled dimension at the nanometer range and to control their spatial distribution and aggregation relies on embedding them into a suitable support or matrix capable of keeping them durably dispersed, thus limiting interparticle magnetic interactions. [20,21,22,23,24,25,26] As a consequence, the magnetic response of the resulting magnetic nanocomposites is due to the degree of control over the monodispersity (in size, shape, and phase) of the nanoparticles population, and to the loading and dispersion of the magnetic phase within the matrix. In particular, magnetic interactions and hardening (or softening) of the magnetic behavior of the nanocomposite is directly influenced by the loading and dispersion of the magnetic phase within the matrix. The capability of obtaining small-sized nanoparticles is of interest because it guarantees that the whole magnetic phase will comprise single domain nanoparticles, i.e., nanoparticles that act as one single magnetic domain under an applied magnetic field, not dividing their atomic moments along different orientations and thus maximizing their overall magnetization.

In this framework, our group has successfully applied a sol-gel route to produce Mn, Zn, Co, Ni, and Cu ferrite nanoparticles which have been crystallized within a porous silica matrix [20,21,22,23,24,25,26]. The sol-gel technique offers the possibility to obtain different forms of porous materials, such as xerogels and aerogels, by adopting specific procedures to obtain the dry gel. It was found that the crystallization of the ferrite nanoparticles within a porous silica matrix represents a valid method to obtain nanocomposites with a homogeneous distribution of the nanoparticles within the matrix, and to limit size growth. In particular, xerogel and aerogel nanocomposites with 10 wt% loading of copper ferrite enabled us to gain insights on the structural properties of CuFe_2_O_4_ nanocrystals, such as the cation distribution within the crystal structure, using X-ray absorption spectroscopy [26]. Interestingly, this study showed that the Jahn–Teller distortion is present, which is consistent with the formation of the tetragonal phase. However, the distortion only interests the copper environment while the iron environment is the same of the disordered cubic phase. Here, we study the magnetic properties of CuFe_2_O_4_-SiO_2_ nanocomposites obtained by sol-gel synthesis in the form of aerogels and xerogels by either supercritical or conventional drying, respectively. The nanocomposites were prepared with ferrite loading ranging between 5 wt% and 15 wt% and submitted to thermal treatments at increasing temperature up to 900 °C. This choice has allowed us to investigate two aspects that come into play in giving rise to the magnetic response of the nanocomposites, namely the presence of small magnetic nanoparticles below the single domain size threshold, and their distribution according to different loadings within matrices with two kinds of porosity.

To perform this study, CuFe_2_O_4_-SiO_2_ aerogel and xerogel nanocomposites with ferrite loading of 5 wt%, 10 wt% and 15 wt% were synthetized and investigated from a structural and magnetometric point of view, to assess the effect of different matrices on the CuFe_2_O_4_ nanoparticles and on the net magnetic features of the nanocomposites.

## 2. Materials and Methods

### 2.1. Synthesis of the Aerogel and Xerogel Nanocomposites

The synthesis of the CuFe_2_O_4_−SiO_2_ aerogel and xerogel nanocomposites follows a two-step catalyzed sol-gel procedure, whose general details are reported in our previously published work [26]. Concisely, a solution of 7.9 mL of tetraethyl orthosilicate (TEOS, Aldrich, UK, 98%) in 3 mL of absolute ethanol (Fischer Chemicals, UK) was pre-hydrolyzed under acidic catalysis through addition of 3.965 mL of a HNO_3_ stock solution prepared by mixing 2 mL of HNO_3_ (Fisher Chemicals, 70%), 80 mL of absolute ethanol, as well as 130 mL of distilled water, and heating at 50 °C for 30 min. After cooling at room temperature, 7.5 mL of an ethanolic solution containing appropriate amounts of Cu(NO_3_)_2_·2.5H_2_O (Aldrich, 98%) and Fe(NO_3_)_3_·9H_2_O (Aldrich, 98%) was added to the TEOS solution. The appropriate amounts of copper and iron nitrates had been calculated in order to obtain nanocomposites with a final CuFe_2_O_4_/(CuFe_2_O_4_+SiO_2_) composition of 5 wt%, 10 wt% and 15 wt%. For the purpose of promoting the condensation reactions and therefore induce gelation, a solution containing 3.513 g of urea (Aldrich, >99%) in 9 mL of absolute ethanol and 4.92 mL of distilled water was added to the TEOS solution, which was then refluxed at 85 °C for 128 min corresponding to the time needed to initiate the gelation. The sol was transferred in vials and left at 40 °C for 20 h to complete gelation.

The aerogels were obtained by ethanol supercritical drying of the gels. For this purpose, the gels were placed in a Parr 300 mL stainless-steel autoclave filled with 70 mL of ethanol and thoroughly flushed with pure N_2_ gas. Once an inert atmosphere was ensured, the sealed autoclave was heated up to 330 °C with a corresponding autogenous pressure of over 70 atm before slowly venting while keeping the temperature constant.

The xerogels were obtained through evaporation of the solvent in an open container at 40 °C for 70 h.

Thermal treatments were performed with a heating ramp of 10 °C·min^−1^ to a variable final temperature followed by a variable dwell time. Aerogel and xerogel samples are indicated, respectively, with “A” or “X” as the first letter in their acronym. The samples have also been labelled with the indication of composition (wt% of CuFe_2_O_4_), temperature and duration of the thermal treatment. For instance, A5_450_1 is used for an aerogel sample with a 5 wt% loading of CuFe_2_O_4_ nanoparticles, thermally treated at 450 °C for 1 h.

### 2.2. Characterization

Powder X-ray diffraction (XRD) patterns were measured by using a Rigaku MiniFlex 600 with a D/teX Ultra high-speed one-dimensional detector (Rigaku, Tokyo, Japan), in the range of 10–90° (2θ), using Cu Kα radiation. The Scherrer equation was used to determine the average size of crystallite domains.

Transmission electron spectroscopy (TEM) images were obtained in bright field (BF) and dark field (DF) modes using a Hitachi H-7000 (Hitachi, Tokyo, Japan) and a Jeol JEM 1400 Plus (Jeol, Tokyo, Japan) equipped with a W thermoionic gun operating at 100 kV respectively and equipped with an AMT DVC (AMT, Danvers, MA, USA) and Ruby2 CCD Camera (Jeol, Tokyo, Japan). 

Magnetic characterization was performed in a Quantum Design MPMS SQUID magnetometer (Quantum Design, San Diego, CA, USA), equipped with a superconducting magnet producing fields up to 50 kOe (5 T). Zero field-cooled (ZFC) and field-cooled (FC) magnetizations were collected in the range of temperatures 5–400 K. ZFC curves were measured by cooling samples in a zero magnetic field and subsequently increasing the temperature under an applied field of 100 Oe. FC curves were recorded by cooling the samples while maintaining the applied field at 100 Oe. Hysteresis loops were recorded up to ±50 kOe (5 T) at 5 K. The samples were placed in gelatin capsules enclosed inside a pierced straw with a uniform diamagnetic background.

Blocking temperature (T_B_) and irreversibility temperature (T_IRR_) indicate the temperature corresponding to the maximum of the ZFC curve and the minimum temperature of superposition between the ZFC and FC curves, respectively. Hysteresis loops were analyzed according to their typical parameters, which are indicated as follows: maximum magnetization (M_5T_ = (|M_+5T_| + |M_−5T_|)/2, where M_+5T_ and M_-5T_ indicate magnetization recorded with and applied field of +/−5 Tesla, respectively), mean remanence (M_R_ = (|M_R+_| + |M_R-_|)/2, where M_R+_ and M_R-_ indicate upper and lower remanence magnetization), mean coercivity (H_C_ = (|H_C1_| + |H_C2_|)/2, where H_C1_ and H_C2_ are the negative and positive coercive fields, respectively) and exchange bias coercivity (H_E_ = (|H_C1_| − |H_C2_|)/2). Saturation magnetization (M_S_) values were determined from the hysteresis loops through extrapolation of M values vs. 1/H for 1/H → 0. All the components of the SQUID samples were weighed for mass of magnetic phase normalization.

## 3. Results and Discussion

**Figure 1:**
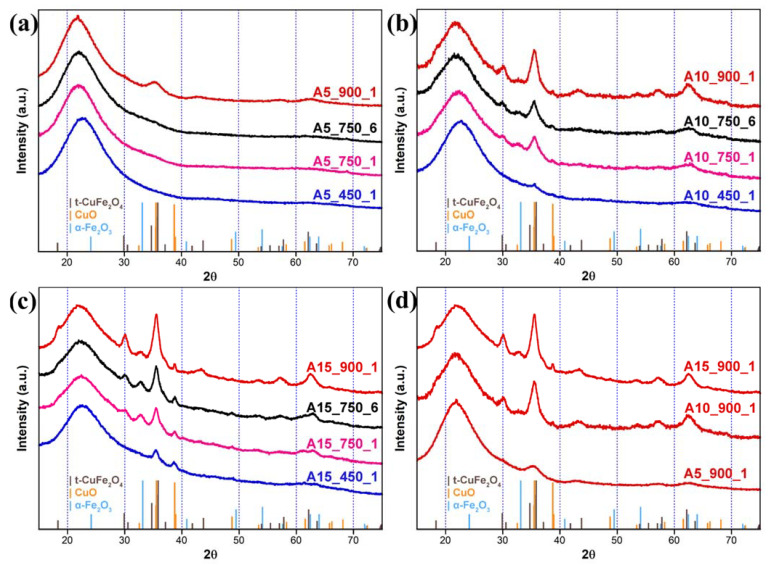
X-ray diffraction (XRD) patterns of the aerogel nanocomposites with (**a**) 5 wt%; (**b**) 10 wt%; (**c**) 15 wt% loading of the dispersed nanophase, reported as a function of the calcination temperature; (**d**) comparison of the XRD patterns of the aerogel samples after thermal treatment at 900 °C, at different CuFe_2_O_4_ loadings.

**Figure 2:**
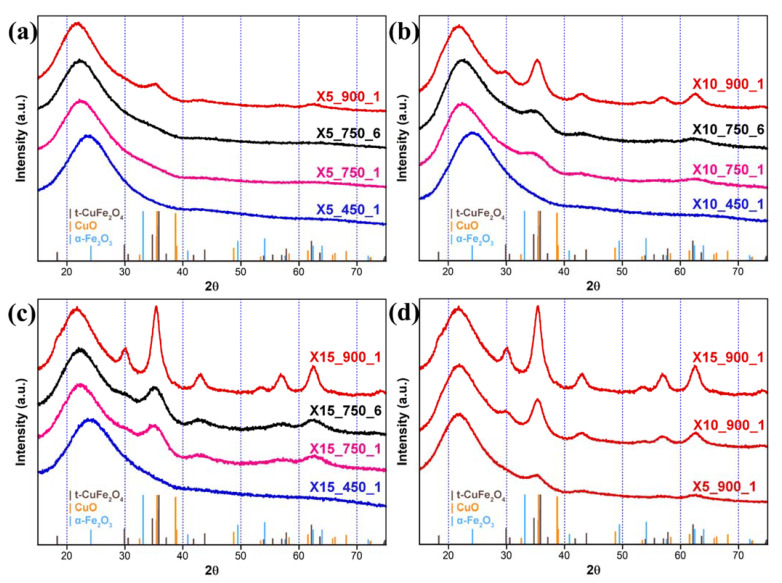
XRD patterns of the xerogel nanocomposites with (**a**) 5 wt%; (**b**) 10 wt%; (**c**) 15 wt% loading of the dispersed nanophase, reported as a function of the calcination temperature; (**d**) comparison of the XRD patterns of the xerogel samples after thermal treatment at 900 °C, at different CuFe_2_O_4_ loadings.

In Figure 1, the powder XRD patterns of the aerogel samples are reported, whereas the XRD patterns of the corresponding xerogel samples are reported in Figure 2. In Figure 1a–c, the XRD patterns compare the evolution of the copper ferrite crystalline phase within the aerogel matrix, as a function of the calcination temperature for any given loading, whereas in Figure 1d, the XRD patterns of the three different loadings are compared after the same thermal treatment at 900 °C. In the case of the 5 wt% nanocomposites (Figure 1a), the XRD pattern is dominated by the presence of the amorphous SiO_2_ phase, highlighted by the typical halo centered at ~22° recurring in all the XRD patterns. Only in the case of the sample treated at 900 °C, some very broad reflections appear, the most intense one centered at ~35.5°, which can be assigned to the formation of either the tetragonal or the cubic crystalline phase of the CuFe_2_O_4_ [27,28]. 

In the case of the aerogels with a 10 wt% and 15 wt% of dispersed phase (Figure 1b,c), some peaks are also detectable in the samples treated at 450 °C and 750 °C, providing some insights on the formation of CuFe_2_O_4_ nanoparticles within the aerogels. In particular, peaks that can be ascribed to CuO [29] are visible in the samples treated at 450 °C, especially in the aerogel with 15 wt% of dispersed phase. As previously found in the sol-gel synthesis of other ferrites dispersed in silica aerogels [30], iron is very likely present in the form of ferrihydrite, whose peaks are hidden within the amorphous silica background because of the poor crystallinity of this phase. When the same samples with a 10 wt% and 15 wt% dispersed phase are submitted to 750 °C some additional peaks appear, which further evolve with increasing the temperature of the thermal treatment. Peaks due to either tetragonal or cubic CuFe_2_O_4_ increase progressively while the peaks due to CuO tend to progressively disappear. Moreover, a peak centered around 33° appears with thermal treatment at 750 °C and then tends to disappear with thermal treatment at 900 °C, indicating the formation of an intermediate phase. Based on the peak position and the composition of the sample, it seems very likely that this peak is due to some hematite forming as intermediate phase from ferrihydrite [31]. The XRD patterns of the samples treated at 750 °C for 1 h and 6 h seem very similar, which proves that thermal treatments longer than 1 h do not induce further crystallization and/or evolution of the initial and intermediate phases. The differences in terms of loading of the crystalline phase are highlighted in Figure 1d, where the intensity of the reflections corresponding to the CuFe_2_O_4_ increases with loading, as was expected.

It should be noted that in the case of the aerogels with a 10 wt% and 15 wt% dispersed phase, two small peaks due to some unreacted α-Fe_2_O_3_ and CuO are still detectable together with the peaks of CuFe_2_O_4_, which appears to be the predominant phase. 

The XRD patterns corresponding to the xerogel samples (Figure 2a–d) show a few differences in the evolution with the thermal treatment, with respect to the aerogel samples. Apart from the typical silica halo, no peaks are detectable in any of the samples treated at 450 °C, regardless of the composition, indicating that very poorly crystalline phases must be present at this stage. When the samples are treated at 750 °C, some peaks appear that become more evident as the loading increases. These peaks are ascribed to the formation of either tetragonal or cubic CuFe_2_O_4_ and further evolve with increasing the temperature of the thermal treatment. The main difference between the xerogel and aerogel samples treated at 900 °C is the width of the peaks, which is larger for the xerogel samples, indicating smaller crystallite sizes. Moreover, in the case of the xerogel samples, all the detectable peaks are due to CuFe_2_O_4_, and no sign of CuO and/or hematite is visible. As mentioned above, the XRD patterns of the nanocomposites do not allow to distinguish between the tetragonal and the cubic crystalline phase of CuFe_2_O_4_ due to the nanocrystalline nature of the samples that generates broad reflections and the XRD patterns of these phases sharing most of the peaks. However, an X-ray absorption investigation on related samples suggested that, under the adopted conditions, CuFe_2_O_4_ nanoparticles crystallize in the tetragonal phase [26]. The crystallite sizes for the samples thermally treated at 900 °C are: 3, 6 and 9 nm for the 5 wt%, 10 wt% and 15 wt% aerogel nanocomposites, respectively, and 3, 4 and 5 nm for the 5 wt%, 10 wt% and 15 wt% xerogel nanocomposites, respectively. Although the sizes increase as a function of the loading, the effect is slower in the xerogel samples versus the aerogel ones.

**Figure 3:**
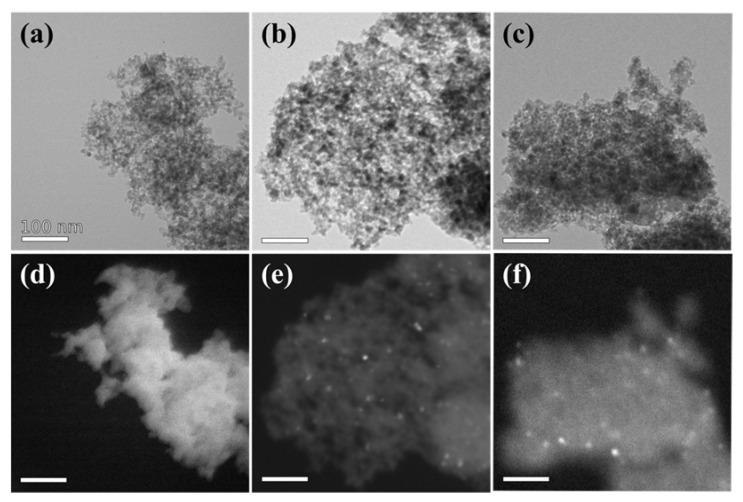
Representative BF (**a**–**c**) and DF (**d**–**f**) transmission electron microscopy (TEM) images of aerogel samples treated at 900 °C with increasing loading of 5 wt% (**a**,**d**), 10 wt% (**b**,**e**) and 15 wt% (**c**,**f**). All scalebars correspond to 100 nm.

**Figure 4:**
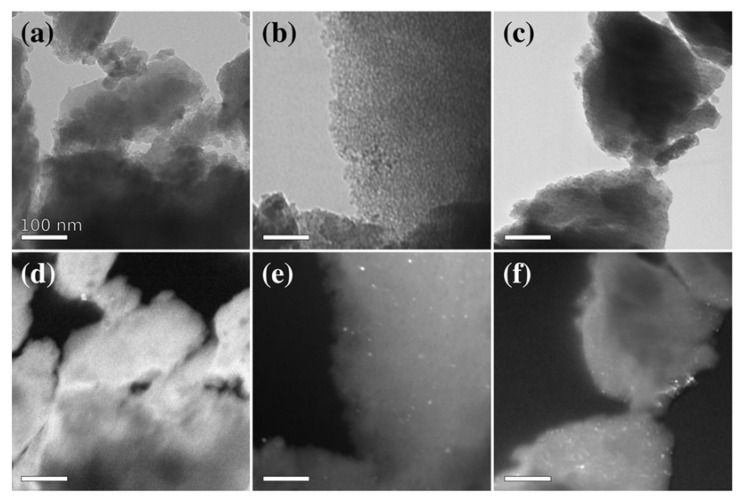
Representative bright field (BF) (**a**–**c**) and dark field (DF) (**d**–**f**) TEM images of xerogel samples treated at 900 °C with increasing loading of 5 wt% (**a**,**d**), 10 wt% (**b**,**e**) and 15 wt% (**c**,**f**). All scalebars correspond to 100 nm.

In Figure 3 and Figure 4, bright-field (BF) and dark-field (DF) images for the aerogel and xerogel samples treated at 900 °C are shown. The images provide clear evidence that the nanoparticles are well dispersed in the SiO_2_ matrix, which is hindering their agglomeration and growth. Their presence can be more clearly observed in the DF images, where the nanoparticles appear as bright dots over a darker background, except for the samples with 5 wt% loading, due to the low amount of ferrite, combined with the very small size of crystallites (3 nm for both samples according to Scherrer calculations).

The images also show a finer and denser texture for the xerogels, as compared to the aerogels, where a more open texture is evident, as was expected [32]. In fact, aerogels obtained via specific gel drying techniques exhibit an open porous network presenting a significant mesopores contribution. On the other hand, xerogels production implies partial collapse of the pores and generates a relatively denser matrix with smaller pores. The size of the nanocrystals increases with ferrite loading and is slightly larger in the aerogel as compared to the xerogel, in agreement with the average nanocrystal size as determined by XRD, likely due to the different matrix characteristics.

DC magnetometry was used to investigate the effect of the ferrite loadings on the overall magnetic properties of the aerogel and xerogel nanocomposites treated at 900 °C, as XRD analysis provides the clearest evidence that nanocrystalline CuFe_2_O_4_ is the most prevalent phase for the samples treated at this temperature. All the relevant parameters are reported in Table 1 and Table 2 for aerogels and xerogels, respectively.

**Figure 5:**
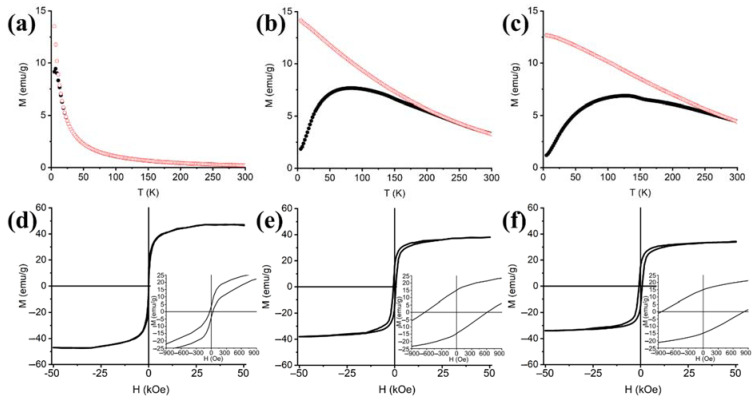
Zero field-cooled–field cooled (ZFC-FC) magnetization curves (**a**–**c**) and hysteresis cycles (**d**–**f**) of aerogel samples treated at 900 °C with increasing loading of 5 wt% (**a**,**d**), 10 wt% (**b**,**e**) and 15 wt% (**c**,**f**).

The three aerogel samples are all magnetically unblocked at room temperature, as shown by ZFC-FC curves (Figure 5a–c) with different T_B_ and T_IRR_ values that tend to increase together with the loading of magnetic phase and crystallite size. Similar trends, i.e., the variation of magnetic parameters with the loading of magnetic phase, can also be appreciated in the hysteresis loops recorded at 5 K (Figure 5d–f). Both the magnetic hardness and the maximum measured magnetization, indicated by coercivity H_C_ and M_5T_, respectively, are affected by the quantity of magnetic phase present in the SiO_2_ matrix. These trends point at a progressive hardening with the increase of Cu ferrite, while the lowered magnetization is consistent with the presence of different crystal phases, as already indicated by the traces of α-Fe_2_O_3_ and CuO observed in the XRD patterns, that are expected to contribute negatively to the net magnetization due to their antiferromagnetic nature. Also, even considering the slight variation in terms of crystallite size between the two samples with highest loadings, the combination of lower magnetization and higher coercivity in the 15 wt% sample with respect to the 10 wt% suggests that a lower number of magnetic moments are available for the reorientation under an applied magnetic field (hence the lower M_S_) and that the said reorientation requires higher applied magnetic fields (hence the higher H_C_).

This finding is in good accordance with the presence of spontaneous exchange bias (H_E_) in the hysteresis loops of the 10 wt% and 15 wt% aerogel samples, whose presence usually indicates structural disorder that depletes the number of available magnetic moments and affects negatively the net magnetization of the samples [33,34,35,36].

On the other hand, the low coercivity, higher magnetization and null exchange bias (H_E_) observed for the 5 wt% sample can be explained by considering the 3 nm size of the crystallites, which puts them in the lower portion of the single domain regime. Such small-sized crystallites correspond to small single magnetic domains, which are easily aligned to applied magnetic fields and undergo superparamagnetic relaxation at very low temperatures, as shown by the low T_B_ and T_IRR_ values observed in the ZFC-FC curves in the present case. Also, no impurities were observed in the corresponding XRD pattern and in this range of sizes no clear distinctions between volume- and surface-based effects should be expected, so the absence of H_E_ and the high values of magnetization can be expected.

**Figure 6:**
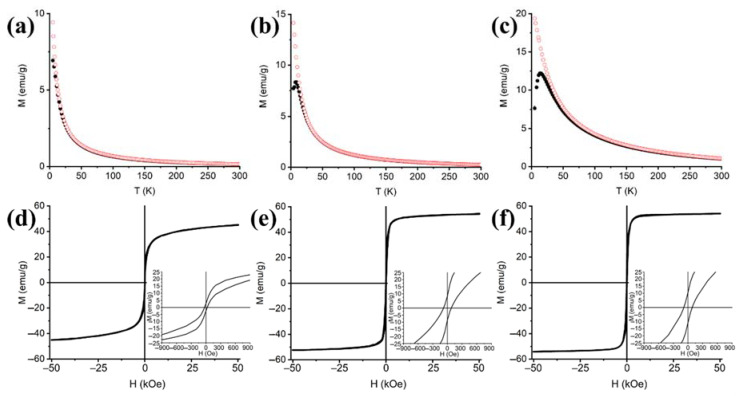
Zero-field cooled-field cooled magnetization curves (**a**–**c**) and hysteresis cycles (**d**–**f**) of xerogel samples treated at 900 °C with increasing loading of 5 wt% (**a**,**d**), 10 wt% (**b**,**e**) and 15 wt% (**c**,**f**).

The magnetic features of the xerogel nanocomposites present some differences from their aerogel counterparts and should be compared in full, taking into account the differences in crystallite size occurring among xerogels as a function of the loading, and between aerogel and xerogel samples with the same loading, as well as the absence of the CuO and α-Fe_2_O_3_ impurities, whose presence was only evidenced in the aerogel samples (Figure 6). At any given loading, blocking and irreversibility temperatures T_B_ and T_IRR_ and coercivity H_C_ have smaller values in the xerogels than in the aerogels. As for the aerogel samples, the lowest percentage of magnetic phase (5 wt%) corresponds to the smallest crystallites, with the xerogel sample having the same average size of the aerogel sample and forming small single magnetic domains that can be easily reoriented along with the external magnetic field. Additionally, spontaneous exchange bias H_E_ is negligible in all the xerogel samples, suggesting a low degree of structural disorder. Since structural disorder can affect the symmetric shape of the hysteresis loops and modify coercivity H_C_ and maximum magnetization values M_5T_ and M_S_, the minimum H_C_ and maximum M_5T_ and M_S_ values observed in xerogel samples are all consistent with the fact that no impurities were observed by XRD in these samples.

The crystal size of the xerogels with 10 wt% and 15 wt% magnetic phase increases slightly with the loading, and it is safe to assume that the magnetic domains follow a similar trend. All the nanoparticles in xerogels also seem less affected by interparticle interactions with respect to the bigger-sized nanoparticles of the aerogels, as indicated by the lower blocking and irreversibility temperatures T_B_ and T_IRR_ observed in the ZFC curves and by their coercivity H_C_. At any given loading, smaller values of all these parameters are observed in the xerogel samples compared to their aerogel counterparts, while their values grow with the loading. For non-interacting nanoparticles, variations in T_B_ and T_IRR_ values are expected, since bigger sizes require increasingly higher temperatures to overcome their blocked state and undergo superparamagnetic relaxation. However, the differences in size between the crystallites of aerogel and xerogel samples cannot solely account for the huge variation observed in the shape and parameters of the ZFC-FC curves. For these reasons, a large effect should be attributed to magnetic interactions affecting the aerogels since these generally work against the easy reorientation of the magnetic moments along an external magnetic field and manifest as a tendency towards magnetic hardening (i.e., higher coercivity) in the hysteresis loops and a broadening in the T_B_ peak of the ZFC curves. The former can be mostly attributed to intraparticle interaction-related structural disorder, while the latter is mostly driven by interparticle interactions [37].

The presence of magnetic interactions in the aerogels can be further proved by comparing the magnetic response of nanoparticles of similar size (6 nm and 5 nm, respectively), present in samples A_10_900_1 and X_15_900_1. Even in this case, despite the lower loading of the aerogel sample A_10_900_1 and the higher defectivity of its crystallites, indicated by the non-null H_E_ value, its T_B_, T_IRR_ and H_C_ values are more than four times higher than those of X_15_900_1, indicating that interparticle magnetic interactions modify the magnetic response of the aerogel samples.

Thermal treatment is known to trigger a progressive reordering of the Cu^2+^ and Fe^3+^ ions in the octahedral and tetrahedral sites, which is proportional to the temperature and clearly modifies the magnetic characteristics of copper ferrite towards soft magnets already at 700 °C [38,39]. In fact, the 900 °C-treated samples are all characterized by higher values of saturation magnetization and lower coercivities than those observed in nanoparticles of similar size that did not undergo post-synthetic thermal treatments [40]. Overall, our DC magnetization studies confirm that the single-domain magnetic nanoparticles included in xerogel matrices are systematically smaller than in their aerogel counterparts and have a lower degree of superficial defectivity. Furthermore, nanoparticles in the xerogels better approximate the typical magnetic response of non-interacting nanoparticles, while magnetic interaction heavily affects the magnetic response of the nanoparticles included in aerogel matrices. In principle, although this difference might be the indication of a higher degree of dispersion of the magnetic nanoparticles in xerogel matrices, given the direct evidence provided by TEM images of the successful homogeneous distribution of the magnetic nanoparticles in both aerogel and xerogel matrices, it should be more likely ascribed to a matrix-based effect on the nanoparticles, which are also influenced by the presence of impurities in the aerogel samples that increase structural disorder in the nanoparticles.

When comparing xerogel and aerogel samples, in addition to the differences in the T_B_ and T_IRR_ values from the ZFC-FC curves, a striking dissimilarity can also be observed with regard to the magnetic hardness in the hysteresis loops recorded at 5 K, i.e., a low temperature where all the samples are in a magnetically blocked state. Here, even taking into account the fact that coercivity increases with the size of the magnetic domain, aerogel samples A10_900_1 and A15_900_1 display coercivity values that are more than five times higher than those of xerogel samples X10_900_1 and X15_900_1 with the same loadings, while also showing non-negligible spontaneous exchange bias H_E_, which is absent for the xerogel samples. Exchange bias manifests in hysteresis loops as a horizontal shift and has been observed in nano-sized compounds with a high degree of superficial structural disorder. Here, the structurally disordered surface of the nanoparticles becomes heavily prone to phenomena such as spin-canting and behaves differently from the structurally ordered core. Then, the system as a whole reacts to applied magnetic fields as a makeshift magnetic core/shell, whose components undergo magnetic coupling during hysteresis loops, giving rise to horizontal shifts that manifest as a difference in the moduli of the negative and positive measured coercive fields, |H_C1_| and |H_C2_|, respectively. When the horizontal shift is observed in zero field-cooled hysteresis loops (such as in the present case), it is usually indicated as spontaneous exchange bias (SEB) [33,34,35,41,42,43,44].

Considering the small size of the magnetic nanoparticles of all the aerogel and xerogel samples, a single domain behavior can be expected in all the nanocomposites, but taking in great care the indications provided by DC magnetometry and XRD, some additional assumptions can be made on both classes of samples. XRD indicates that the nanoparticles of A10_900_1 and A15_900_1 are bigger than those of X10_900_1 and X15_900_1, and also shows the presence of a few low intensity peaks that are attributed to impurities of CuO and α-Fe_2_O_3_. While the formation of small impurities is not expected to revolutionize the overall magnetic response of A10_900_1 and A15_900_1, their presence paired with increased magnetic hardness, spontaneous exchange bias and higher unblocking temperatures suggest that the magnetic nanoparticles included in the aerogel matrices tend to be more structurally disordered and that the presence of impurities might be located at the nanoparticles’ surface, thus increasing the superficial disorder and consequently giving rise to a patched makeshift shell from the structural point of view. If one considers these “composed” nanoparticles, it is clear why they require higher temperatures to undergo superparamagnetic relaxation and can reach lower saturation magnetization: the structural disordered surface lowers the number of atomic moments that can be successfully reoriented under an external magnetic field, while opposing the thermal unblocking and the re-orientation of magnetic moments from the ordered “core” region. On the other hand, the lower unblocking temperatures and the narrow, symmetric hysteresis loops indicate that the xerogel samples contain smaller, well-formed crystal domains that do not show the disorder-related responses observed in aerogel samples and give rise to nanocomposites with a softer magnetic response and higher saturation magnetization.

Even considering the varied effect of the xerogel and aerogel matrices on the final magnetic features of the nanocomposites and the minor impurities observed in the aerogel samples, the controlled inclusion of copper ferrite nanoparticles in both xerogel and aerogel affects their magnetic features and distances them from the magnetic response of a typical population of similar sized unsupported nanoparticles [40,45]. In a comparison between evenly sized unsupported nanoparticles and nanoparticles included in nanocomposites, the former are magnetically harder, with higher exchange bias and tendentially lower saturation magnetization values. This means that the inclusion of copper ferrite nanoparticles inside aerogel and xerogel matrices helps in controlling their size and maintaining it well within the single domain regime, lowers the interparticle interaction and consequently boosts the saturation magnetization, while restoring the soft magnetic features typically observed in copper ferrite.

## 4. Conclusions

The sol-gel method used has shown to be effective in obtaining nanocomposites where copper ferrite nanoparticles are well dispersed in a silica matrix, both in the form of a highly porous aerogel or a denser xerogel. Crystallization of the copper ferrite phase within the matrix is achieved through thermal treatments of the xerogels and aerogels, and samples exhibit nanoparticles with average size within 10 nm after treatment at 900 °C. Even at this high temperature the nanocomposites maintain their porous structure as evidenced by the TEM images, with nanoparticles which are very well separated within the matrix. The average sizes of the ferrite nanoparticles depend on both the texture of the matrix and the loading of the ferrite, influencing the overall magnetic behavior of the samples. In particular, DC magnetometry indicates that the samples exhibit a superparamagnetic behavior and are unblocked at room temperature, with magnetic interactions partially coming into play in determining the final magnetic features of the nanocomposites. Moreover, the results obtained by DC magnetometry at low temperature, when the nanocomposites are in a magnetically blocked state, highlight how the spatial distribution of the magnetic phase operated by aerogel and xerogel matrices helps in boosting the saturation magnetization and lowering the coercivity in comparison with the magnetic response obtained by populations of unsupported nanoparticles of similar size.

## Figures and Tables

**Table 1:** Magnetic parameters for the 5 wt%, 10 wt% and 15 wt% CuFe_2_O_4_-SiO_2_ nanocomposite aerogel samples. Relevant parameters are indicated as T_B_ (blocking temperature), T_IRR_ (irreversibility temperature), H_C_ (coercivity), H_E_ (exchange bias), M_R_ (remanent magnetization, M_5T_ (maximum magnetization, M_S_ (saturation magnetization). Hysteresis loops were recorded at 5 K. Values were determined according to what was reported in the Materials and Methods section.

Sample	T_B_ (K)	T_IRR_ (K)	H_C_ (Oe)	H_E_ (Oe)	M_R_ (emu/g)	M_5T_ (emu/g)	M_S_ (emu/g)	M_R_ /M_S_	M_5T_ /M_S_
A5_900_1	7	13	32	1	4	47	51	0.08	0.93
A10_900_1	83	225	623	18	15	38	39	0.38	0.97
A15_900_1	127	294	825	12	15	34	34	0.41	0.94

**Table 2:** Magnetic parameters for the 5 wt%, 10 wt% and 15 wt% CuFe_2_O_4_ -SiO_2_ nanocomposite xerogel samples. Relevant parameters are indicated as T_B_ (blocking temperature), T_IRR_ (irreversibility temperature), H_C_ (coercivity), H_E_ (exchange bias), M_R_ (remanent magnetization, M_5T_ (maximum magnetization, M_S_ (saturation magnetization). Hysteresis loops were recorded at 5 K. Values were determined according to what was reported in the Materials and Methods section.

Sample	T_B_ (K)	T_IRR_ (K)	H_C_ (Oe)	H_E_ (Oe)	M_R_ (emu/g)	M_5T_ (emu/g)	M_S_ (emu/g)	M_R_ /M_S_	M_5T_ /M_S_
X5_900_1	5	11	28	1	9	45	53	0.16	0.85
X10_900_1	9	32	80	0	9	54	58	0.15	0.93
X15_900_1	17	53	128	0	13	51	51	0.25	0.99

## Data Availability

The data presented in this study are available on request from the corresponding author.
